# New Antipyretics and Proprietary Medicines

**Published:** 1888-08

**Authors:** 


### NEW ANTIPYRETICS, AND PROPRIETARY MEDICINES.

Until the recent great advances in chemistry and pharmacology gave us the revelations of the laboratory, it seemed as if we were not greatly wanting in antipyretics in the physician's armamentarium, ranging from cold baths to venesection. The introduction of antipyrin and antifebrin and other recent antipyretics seems at present to have almost supplanted those on which implicit reliance had been placed. Will their present claims stand the crucial test of time? Will they prove as free from danger, as safe, as they are now believed to be?

Their introduction has raised another question—one regarding proprietary medicines—a question that has not been wholly decided yet by the great body of physicians in this country. Not unnaturally, American manufacturers of proprietary medicines are asking what makes the difference between proprietary medicines manufactured on the two sides of the Atlantic. Shall American physicians prescribe only such as have been manufactured on the eastern side of that great ocean, and, if so, for what reason? The same objection holds good against all to which a name is given which conveys little, if any, idea of the component parts. Only careful analysis can determine whether published formulæ indicate reliably their ingredients, but the same is equally true, in some respects, of many of the alleged officinal preparations.

It must be conceded that many of the manufacturing chemists, at home and abroad, are furnishing us with preparations that are elegant in form, and apparently quite as reliable in composition as many of the officinal preparations that are dispensed, and having a great advantage in being less unpalatable. If sufficient guaranty can be given the prescriber that he can as safely rely upon the accuracy of the published formulæ of these unofficinal as upon the officinal preparations, it is not improbable that they will in future be prescribed with increasing frequency by a large part of the profession, who believe that the active competition between rival manufacturing chemists and the advance in chemistry and pharmacy, have resulted in the production of many preparations of merit, which until recently were wanting.

It is not essential that the names of any of the well-known manufacturing chemists be cited to illustrate the wide difference which exists between them as manufacturers of proprietary medicines and others who are engaged in concocting the nostrums that are vended and popularly known as patent medicines. Their methods of disposing of their productions differ as widely as their products, and although they seek to protect by legal methods the results of their skill and monetary expenditure in a manner that does not obtain in the profession of medicine, the question seems to have been settled, at least by usage, that that fact alone shall not preclude their being prescribed by members of the regular profession on the ground that they are regarded in the light of "patent or secret medicines" as specified in our code of ethics.

If the better class of manufacturing chemists continue to place their products upon the market with the conscientious regard for truth and propriety which has characterized their course in the past, and shall not allow competition to tempt them into the methods by which nostrum-venders seek to dispose of their wares, there need be no more cause for classifying them in the same category than of doing so with the educated and meritorious physician and the blatant, ignorant, and pretentious mountebank.

